# Supplementary material for: Association of Standardized Parenteral Nutrition with Early Neonatal Growth of Moderately Preterm Infants: A Population-Based Cohort Study
Source: Nutrients. 2024 Apr 26;16(9):1292. doi: 10.3390/nu16091292 (PMC11085250; doi:10.3390/nu16091292)
Supplement: Supplementary file 1 [file nutrients-16-01292-s001.zip › nutrients-2971212-supplementary.pdf]

**Table S1:** Composition of 3-in-1 standardized parenteral nutrition solutions.

| | Solution 1 | Solution 2 | Solution 3 |
|---|---|---|---|
| **Volume, mL** | 250 | 400 | 400 |
| **Osmolarity, mOsm/L** | 714 | 780 | 960 |
| **Per 100 ml** | | | |
| **Total calories, kcal** | 49 | 61 | 74 |
| **Glucose, g** | 8 | 9 | 10.7 |
| **Amino acids, g** | 2 | 2.2 | 2.7 |
| **Lipids, g** | 1 | 1.8 | 2.2 |
| **Sodium, mmol** | 2 | 2.2 | 2.5 |
| **Potassium, mmol** | 2 | 1.4 | 1.7 |
| **Magnesium, mmol** | 0.3 | 0.2 | 0.2 |
| **Calcium, mmol** | 1.3 | 0.8 | 1.2 |
| **Phosphate, mmol** | 1 | 1 | 1.2 |
| **Chloride, mmol/l** | 1.6 | 1.6 | 2.1 |
| **Carnitine, mg** | 8 | 8 | 5.7 |
| **Vitamin A, IU** | 460 | 460 | 320 |
| **Vitamin $D_2$, IU** | 80 | 80 | 56 |
| **Vitamin E, IU** | 1.4 | 1.4 | 1 |
| **Phytomenadione, µg** | 40 | 40 | 28 |
| **Ascorbate, mg** | 11 | 11 | 8 |
| **Thiamine, µg** | 250 | 250 | 175 |
| **Riboflavine, µg** | 360 | 360 | 250 |
| **Pyridoxine, µg** | 400 | 400 | 280 |
| **Nicotinamide, mg** | 4 | 4 | 2.8 |
| **Pantothenate, mg** | 1.6 | 1.6 | 1.1 |
| **Biotin, µg** | 6 | 6 | 4 |
| **Folate, µg** | 40 | 40 | 25 |
| **Cyanocobalamine, µg** | 0.5 | 0.5 | 0.3 |
| **Zinc, µg** | 60 | 100 | 315 |
| **Copper, µg** | 12 | 20 | 13 |
| **Manganese, µg** | 0.3 | 0.5 | 0.3 |
| **Iodine, µg** | 0.6 | 1 | 0.6 |
| **Selenium, µg** | 1.2 | 2 | 1.3 |
